# Distribution of Helminth Parasites in Intestines and Their Seasonal Rate of Infestation in Three Freshwater Fishes of Kashmir

**DOI:** 10.1155/2016/8901518

**Published:** 2016-09-21

**Authors:** Asifa Wali, Masood-ul Hassan Balkhi, Rafia Maqbool, Mohammed Maqbool Darzi, Feroz Ahmad Shah, Farooz Ahmad Bhat, Bilal Ahmad Bhat

**Affiliations:** ^1^Faculty of Fisheries, Sher-e-Kashmir University of Agricultural Sciences and Technology of Kashmir, Rangil, Ganderbal, Jammu and Kashmir 19006, India; ^2^Division of Veterinary Microbiology, Faculty of Veterinary Sciences and Animal Husbandry, Sher-e-Kashmir University of Agricultural Sciences and Technology, Shuhama, Jammu and Kashmir 19006, India; ^3^Division of Veterinary Pathology, Faculty of Veterinary Sciences and Animal Husbandry, Sher-e-Kashmir University of Agricultural Sciences and Technology, Shuhama, Jammu and Kashmir 19006, India

## Abstract

The present study was undertaken to determine the incidence of helminth parasites in fishes with special reference to water quality parameters in Dal Lake and River Jhelum and correlate the observations. Water, fish, and parasite samples were collected during different seasons from various sites and processed. Three fish species, namely,* Schizothorax niger *Heckel 1838,* Schizothorax esocinus *Heckel 1838, and* Schizothorax curvifrons* Heckel 1838, were recovered from these water bodies. The physicochemical parameters temperature, dissolved oxygen, pH, and free carbon dioxide showed variation vis-à-vis the season and location of the stations in water bodies. Acanthocephalan parasite* Pomphorhynchus kashmirensis *Kaw 1941 (27.47%) and two intestinal cestodes* Bothriocephalus acheilognathi *Yamaguti 1934 (30.63%) and* Adenoscolex oreini *Fotedar 1958 (32.43%) were recovered from all the three species of* Schizothorax*. All the three parasites showed higher prevalence during summer and the least prevalence during winter. Parasitic infections were prevalent more in male fishes compared to females. The presence of the parasites had reduced the condition coefficient of the infected fishes in both water bodies. The study also showed that some of the physicochemical features showed a significant positive correlation with the prevalence.

## 1. Introduction

Jammu and Kashmir is gifted with water resources of about 40,000 ha comprising lakes, streams, rivers, springs, and so forth suitable for fish culture. Aquaculture is one of the most economically important applied strategies all over the world and fishes are one of the most beneficial and nutritional resources of human beings. The aquatic environment of fresh water resources encompasses a wide variety of features, namely, physicochemical, biological, and ecological characteristics, virtually all of which influence the maintenance of homeostasis, growth, and reproduction of fish [1, 2]. The environmental factors are never constant; they fluctuate and keep stresses on organisms. These environmental alterations influence organisms physiologically in various ways. They may be lethal, modifying the effect of some other factors, directive, or controlling. The same abiotic environmental factors may produce different effects at different times and under different conditions, and if these features are altered beyond acceptable limits, they may cause a wide range of diseases in fish [3–5]. Fishes are hosts to a number of parasites. Helminths are one of the major groups of fish parasites and cause a severe loss in the fish production [6, 7]. Fishes are infected with three major groups of helminths: the Platyhelminthes (flat worms), Nematoda (round worms), and Acanthocephala (spiny headed worms). About 20,000 to 30,000 helminth species have been reported worldwide, which cause heavy losses to the fish industry [8]. Dhar (1972) [9] reported 31 species of helminth parasites from Kashmir valley which cause severe damage to the fish production and population. The present study was undertaken to study the incidence of helminth parasites in fishes with special reference to water quality parameters and to correlate the parasitic prevalence and various physicochemical parameters.

## 2. Materials and Methods

### 2.1. Physicochemical Parameters of Water

Water samples were collected during each survey and analyzed for various physicochemical parameters like water temperature, dissolved oxygen, free carbon dioxide, and pH. The collected samples were analyzed according to APHA (2005) [10] for different physicochemical parameters. Temperature was recorded by using a mercury filled thermometer and results were expressed as °C. pH of the water was determined with the help of conductivity meter. Free carbon dioxide was measured by using phenolphthalein indicator and sodium hydroxide titrant. For dissolved oxygen, samples were fixed on the spot as per Winkler's unmodified method and brought into the laboratory for further detailed analysis.

### 2.2. Examination of Fish for Helminth Parasitic Infestation

Three fish species of schizothoracine (*Schizothorax niger* Heckel 1838,* Schizothorax esocinus* Heckel 1838, and* Schizothorax curvifrons *Heckel 1838) were collected on monthly basis and carried to the laboratory in plastic bags. Every effort was made to keep them alive. After giving them serial number, morphometric characters including total length, fork length, total weight, and sex were determined. The fishes were killed by severing the spinal cord behind the head and were subsequently dissected by making an insertion from the anus towards the head. Once they had been dissected, the intestines were removed and placed in a normal saline solution in Petri dishes for examination. Parasites were collected as soon as possible after the death of the fish to prevent any deterioration. The intestines were pulled open carefully using two sharp tweezers to ensure that the cestodes were kept intact. Each cestode was carefully and slowly dislodged from the intestinal wall, ensuring that it remained intact. They were transferred to a clean sampling bottle containing normal saline solution, which was then shaken vigorously for a few minutes to dislodge debris and induce muscle fatigue in the helminths, which in turn deters strong contraction of the scolices and relaxes them. While swirling the sampling bottle, an equal amount (equal to the amount of saline solution already present in the sampling bottle) of a hot alcohol-formaldehyde-acetic acid (AFA) solution was added to kill and fix the specimens. Specimens were then stored in 70% alcohol. The cestodes were stained with Grenacher's borax carmine stain [11] and identified. Acanthocephalans were removed from the host without any form of treatment prior to preservation except that acanthocephalans were relaxed in tap water so that specimens with proboscis fully everted were produced. In case the anterior end was deeply bored in the mucosa of the intestine, a few crystals of methanol were added to the normal saline, containing the parasites adhered to the intestinal wall. This led to immobilization of the parasites and loosening of the grip on the intestinal wall and facilitated the detachment of proboscis in case of acanthocephalans without causing any distortion in the arrangement of hooks.

### 2.3. Identification of Parasites

The parasitological examination of fishes was carried out as per the methodology of [12]. The parasites were processed and identified with the help of keys provided by [13–16]. The prevalence, mean intensity, and relative density of helminth parasites were calculated in accordance with [17, 18]. The data collected was statistically analyzed using SPSS version 20 software. Data were expressed as mean ± SD and significant correlation and chi square tests were implied wherever necessary.

## 3. Results

### 3.1. Physicochemical Parameters

The present study showed that physicochemical parameters did not remain stable for a prolonged period at a particular place and show fluctuations from region to region and season to season. Marked differences were observed in the two water bodies during the study period (Tables 1 [Table tab2] [Table tab3]–4). There was an increasing trend from winter to summer in water temperature in all the sites. The minimum and maximum temperatures recorded in different stations during different seasons ranged from 4 to 27°C. The dissolved oxygen concentrations during autumn and summer were significantly (*p* < 0.05) lower than those in spring and winter seasons. The pH value was the highest during summer and the lowest during autumn. The maximum pH value recorded was 8.4 during summer and the minimum was 7.03 during autumn. The minimum free carbon dioxide concentration was 3.80 ± 0.92 mg/L during autumn whereas the maximum concentration was 7.52 ± 2.84 mg/L during spring season.

### 3.2. Levels of Infections in Fishes

A total of three helminth parasitic species were recovered from 444 examined specimens of* Schizothorax* spp. 122 (27.47%) were found to harbor the* Pomphorhynchus kashmirensis*, 136 (30.63%) were found to harbor the* Bothriocephalus acheilognathi *parasite, and 144 (32.43%) were found to be infected with the* Adenoscolex oreini.*

### 3.3. Fish Species-Wise Prevalence

224 specimens were examined from the Dal Lake. Only 47 specimens were found to be infected with* Pomphorhynchus kashmirensis* (20.98%), which showed distribution of the parasite in* S. niger*,* S. esocinus*, and* S. curvifrons* (27.63, 18.18, and 16.90%, resp.) which varied significantly (*p* < 0.01). 220 specimens were examined from River Jhelum. Only 75 (34.07%) were infected with* Pomphorhynchus kashmirensis* which include* S. niger* (30.20%),* S. esocinus* (30.13%), and* S. curvifrons* (42.25%) (Table 5).

224 fishes were examined from the Dal Lake. Only 63 (28.14%) were found to be infected with* Bothriocephalus acheilognathi*. Fish-wise distribution of the parasite was highly significant (*p* < 0.01) which showed* S. niger* (28.94%),* S. esocinus* (31.16%), and* S. curvifrons* (23.94%). Out of 220* Schizothorax* spp. from River Jhelum, 73 (33.18%) were infected with* Bothriocephalus acheilognathi* which include* S. niger* (34.21%),* S. esocinus* (34.24%), and* S. curvifrons* (30.98%) (Table 8).

224 specimens were examined from the Dal Lake. 71 (31.69%) specimens were found to be infected with* Adenoscolex oreini*. Fish-wise distribution of the parasite was significantly varied (*p* < 0.01) which showed* S. niger* (31.57%),* S. esocinus* (28.57%), and* S. curvifrons* (35.21%). Out of the 220* Schizothorax* spp. examined from River Jhelum, 73 (33.18%) were infected with* Adenoscolex oreini* which included* S. niger* (28.94%),* S. esocinus* (32.87%), and* S. curvifrons* (38.02%) (Table 11).

### 3.4. Seasonal Prevalence of Helminths

Seasonal prevalence of* Pomphorhynchus kashmirensis*,* Bothriocephalus acheilognathi*, and* Adenoscolex oreini *infection showed a definite trend. The infection was the highest in summer and the lowest in winter. There was a gradual increase in the prevalence rate from spring to summer which fell down with onset of autumn and later on was least observed during winter season (Tables 6, 9, and 12).

### 3.5. Fish Gender-Wise Prevalence

All the three parasitic infections were prevalent more in male fishes compared to females. In Dal Lake, the overall prevalence of* Pomphorhynchus kashmirensis*,* Bothriocephalus acheilognathi*, and* Adenoscolex oreini *was 23.1%, 31.34%, and 32.83%, respectively, in males whereas it was 17.7%, 23.3%, and 30%, respectively, in females. In River Jhelum, the overall prevalence in males was 35.8%, 41.02%, and 36.75%, respectively, while in females it was 32.03%, 24.29%, and 23.3%, respectively (Tables 7, 10, and 13).

### 3.6. Influence of Sex and Condition Factor on the Level of Infection

An insignificant relationship existed between gender and helminth infection. Condition factors were found to be lower in infected fish than in uninfected fish in both water bodies.

The analysis of condition factor by Mann-Whitney test revealed* S. niger* (*U* = 13, *p* < 0.01),* S. esocinus* (*U* = 45, *p* > 0.05), and* S. curvifrons* (*U* = 34, *p* > 0.05) of Dal lake, while in River Jhelum it revealed* S. niger* (*U* = 3, *p* < 0.01),* S. esocinus* (*U* = 3, *p* < 0.01), and* S. curvifrons* (*U* = 16, *p* < 0.05). Analysis of the condition factor of uninfected and infected* Schizothorax* spp. of Dal Lake and River Jhelum revealed significant differences (*p* < 0.05) with higher values in River Jhelum.

### 3.7. Correlation between Prevalence and Water Quality in Two Water Bodies (Table 14)

Temperature was the most important abiotic factor that affected the parasites at all life cycle stages. A positive correlation (*p* < 0.01) existed between water temperature and parasitic prevalence in Dal Lake and River Jhelum.

Prevalence of* P. kashmirensis* and* A. oreini* in the fishes of Dal Lake presented a significant negative correlation (*p* < 0.01) with dissolved oxygen whereas it showed an insignificant negative correlation (*p* > 0.05) in all other cases of patterns of infection under various locations.

pH showed an insignificant positive correlation (*p* > 0.05) with all parasitic infections. Prevalence of infections showed an insignificant positive correlation (*p* > 0.05) with carbon dioxide except for* P. kashmirensis* of River Jhelum and* B*.* acheilognathi* of Dal Lake and River Jhelum which showed a significant positive correlation (*p* < 0.05).

## 4. Discussion

The temperature of water in Jhelum River is at lower degree than that of Dal Lake which might be attributed to the flowing nature of water in Jhelum. Water temperature in summer was high due to low water level, high atmospheric temperature, and clear atmosphere [19]. The fluctuation in the DO value might be due to differences in water temperature [20]. The desirable limit for pH is 6.0 to 8.0; however, some sites crossed the desirable limit. The fluctuations might be due to low rates of decomposition and good amount of calcium carbonates and magnesium in the area. Moreover, due to the greater photosynthetic activity, greater utilization of CO_2_ is responsible for increased pH (alkaline) [21, 22]. Free carbon dioxide was found to be higher in Dal Lake than in River Jhelum in all the seasons which may be due to alkalinity and hardness of the water body. The value of CO_2_ was high in spring and summer. The increasing trend of free carbon dioxide down the river could be due to the addition of some carbon rich substances as majority of carbon comes from organic matter such as ground water, rock leaching, and dead terrestrial plant material [23].

Infection patterns of* Pomphorhynchus *were greatly influenced by season, fish species, and type of water body. It was seen that overall prevalence of* Pomphorhynchus *was low, compared to the other two helminthes. The low prevalence might be due to low availability or consumption of intermediate hosts. For both cestodes, clear seasonal trend was observed in Dal Lake and River Jhelum with maximum infection level during summer months and the least level in winter months. Significant differences (*p* < 0.01) in prevalence were recorded vis-à-vis the season in both water bodies which were in conformity with the results of [24] that concluded that the helminth species like monogeneans showed seasonal alterations associated with environmental changes. The abrupt increase in helminth infection from summer in both water bodies could be due to increased duration of life of the infective larva and has been reported to assist in the transfer of helminth infection like* Diplozoon* infection from fish to fish [25]. Both the cestodes and the acanthocephalan infection were prevalent more in male fishes compared to females. Takemoto and Pavanelli (2000) [26] reported that male hosts had significantly higher parasite intensity than females. The influence of sex on the susceptibility of animals to infections could be attributed to genetic predisposition and differential susceptibility owing to hormonal control. Condition coefficient was found to be lower in infected fish in both Dal Lake and River Jhelum, which might be due to the fact that parasites decrease the immune system of the hosts, which may lead to decreased growth of fish. Decreased growth may lead to a decrease in condition coefficient [27, 28]. Physicochemical features showed a significant positive correlation with the prevalence. Modu et al. (2011) [29] showed that there existed a significant correlation between helminth infection and water quality parameters in a pond. A number of workers [30–33] have suggested that natural abiotic factors such as temperature, oxygen, salinity, hydrogen ion concentration, and eutrophication have a positive influence on the occurrence of parasite populations.

## Figures and Tables

**Table 1:** Seasonal mean temperatures (°C) of the water samples collected from different sites of Dal Lake and River Jhelum.

Location	Seasons				Overall mean	CD
	Autumn (mean ± SD)	Winter (mean ± SD)	Spring (mean ± SD)	Summer (mean ± SD)		
*Dal Lake*						
Dalgate	18.03 ± 4.08	6.55 ± 1.48	17.88 ± 2.97	**27.22 ± 1.71**	17.42 ± 8.45	**2.67**
Saida Kadal	17.07 ± 4.18	6.77 ± 1.85	19.66 ± 3.60	25.88 ± 1.36	17.35 ± 7.96	**2.88**
Hazratbal	19.24 ± 5.46	7.04 ± 2.89	18.27 ± 2.92	26.55 ± 2.45	17.78 ± 8.05	**3.50**
Telbal	17.22 ± 2.88	6.68 ± 2.20	17.55 ± 3.12	25.33 ± 2.34	16.70 ± 7.65	**2.57**
*River Jhelum*						
Chattabal Weir	14.16 ± 2.91	4.77 ± 1.39	14.00 ± 3.90	19.44 ± 1.50	13.09 ± 6.09	**2.55**
Zerobridge	15.44 ± 2.66	5.33 ± 1.32	14.00 ± 3.84	18.88 ± 1.26	13.41 ± 5.76	**2.88**
Khannabal	13.50 ± 3.39	4.37 ± 1.32	13.00 ± 3.39	18.00 ± 1.32	12.21 ± 5.69	**2.88**

*Overall mean*	**16.38 ± 2.08**	**5.93 ± 1.08**	**16.34 ± 2.60**	**23.04 ± 4.05**	**15.42 ± 2.40**	
*CD*	Location = 1.32					
	Seasons = 1.00					
	Location × seasons = 2.64					

**Table 2 tab2:** Seasonal mean dissolved oxygen (mg/L) of the water samples collected from different sites of Dal Lake and River Jhelum.

Location	Seasons				Overall mean	CD
	Autumn (mean ± SD)	Winter (mean ± SD)	Spring (mean ± SD)	Summer (mean ± SD)		
*Dal Lake*						
Dalgate	4.18 ± 0.38	5.87 ± 1.27	5.35 ± 0.73	4.14 ± 1.32	4.89 ± 0.86	**0.97**
Saida Kadal	4.21 ± 1.55	5.57 ± 1.10	4.84 ± 1.02	4.28 ± 1.03	4.73 ± 0.63	**NS**
Hazratbal	4.20 ± 1.22	5.67 ± 0.88	5.28 ± 1.70	3.55 ± 1.19	4.68 ± 0.97	**1.24**
Telbal	5.41 ± 1.38	6.73 ± 1.13	6.78 ± 1.25	5.80 ± 1.15	6.18 ± 0.68	**NS**
*River Jhelum*						
Chattabal Weir	5.58 ± 1.50	6.16 ± 0.79	6.28 ± 0.93	4.98 ± 1.28	5.75 ± 0.59	**NS**
Zerobridge	4.97 ± 1.50	5.76 ± 0.66	6.72 ± 1.44	5.41 ± 1.38	5.71 ± 0.74	**NS**
Khannabal	5.86 ± 1.66	6.26 ± 1.13	8.16 ± 1.43	6.37 ± 1.42	6.66 ± 1.02	**NS**

*Overall mean*	4.92 ± 0.72	6.01 ± 0.40	6.20 ± 1.14	4.93 ± 1.00	5.51 ± 0.78	
*CD*	Location = 0.57					
	Seasons = 0.43					
	Location × seasons = NS					

**Table 3 tab3:** Seasonal mean pH of the water samples collected from different sites of Dal Lake and River Jhelum.

Location	Seasons				Overall mean	CD
	Autumn (mean ± SD)	Winter (mean ± SD)	Spring (mean ± SD)	Summer (mean ± SD)		
*Dal Lake*						
Dalgate	7.45 ± 0.35	7.87 ± 0.39	7.60 ± 0.26	8.31 ± 0.42	7.81 ± 0.37	**0.35**
Saida Kadal	7.93 ± 0.50	8.04 ± 0.48	8.16 ± 0.36	8.14 ± 0.40	8.07 ± 0.10	**NS**
Hazratbal	8.20 ± 0.61	8.23 ± 0.34	8.42 ± 0.43	8.47 ± 0.41	8.33 ± 0.13	**NS**
Telbal	7.63 ± 0.43	7.83 ± 0.25	7.65 ± 0.44	7.86 ± 0.35	7.74 ± 0.12	**NS**
*River Jhelum*						
Chattabal Weir	7.03 ± 0.28	7.49 ± 0.40	7.67 ± 0.32	7.87 ± 0.30	7.52 ± 0.35	**0.48**
Zerobridge	7.20 ± 0.59	7.97 ± 0.43	8.10 ± 0.48	7.85 ± 0.41	7.78 ± 0.40	**0.51**
Khannabal	7.84 ± 0.52	7.94 ± 0.80	8.35 ± 0.51	8.07 ± 0.40	8.05 ± 0.22	**NS**

*Overall mean*	7.61 ± 0.41	7.91 ± 0.22	7.99 ± 0.34	8.08 ± 0.24	7.90 ± 0.24	
*CD*	Location = 0.20					
	Seasons = 0.15					
	Location × seasons = 0.41					

**Table 4:** Seasonal mean free carbon dioxide (mg/L) of the water samples collected from different sites of Dal Lake and River Jhelum.

Location	Seasons				Overall mean	CD
	Autumn (mean ± SD)	Winter (mean ± SD)	Spring (mean ± SD)	Summer (mean ± SD)		
*Dal Lake*						
Dalgate	3.94 ± 0.72	3.38 ± 1.39	7.11 ± 1.69	6.88 ± 3.37	5.33 ± 1.94	**1.97**
Saida Kadal	3.16 ± 0.93	5.55 ± 1.42	9.50 ± 3.33	7.55 ± 7.46	6.44 ± 2.71	**4.03**
Hazratbal	5.77 ± 1.20	6.43 ± 1.31	5.10 ± 3.84	3.55 ± 1.23	5.21 ± 1.23	**NS**
Telbal	3.83 ± 1.00	5.22 ± 1.48	12.88 ± 7.21	5.44 ± 2.69	6.84 ± 4.09	**3.81**
*River Jhelum*						
Chattabal Weir	3.08 ± 1.89	4.61 ± 2.52	7.33 ± 1.41	8.11 ± 2.26	5.78 ± 2.34	**2.19**
Zerobridge	3.27 ± 0.90	3.55 ± 1.57	4.84 ± 1.00	8.05 ± 6.28	4.93 ± 2.19	**3.32**
Khannabal	3.55 ± 1.33	3.92 ± 0.95	5.88 ± 3.68	8.44 ± 5.43	5.45 ± 2.24	**3.40**

*Overall mean*	3.80 ± 0.92	4.67 ± 1.12	7.52 ± 2.84	6.86 ± 1.77	5.71 ± 2.39	
*CD*	Location = NS					
	Seasons = 1.10					
	Location × seasons = 2.91					

**Table 5:** Overall prevalence of *Pomphorhynchus kashmirensis *in various host species.

Host	Dal Lake							River Jhelum						
	Number examined	Number infected	Prevalence (%)	Number of parasites	Mean intensity	Abundance	*p* value	Number examined	Number infected	Prevalence (%)	Number of parasites	Mean intensity	Abundance	*p* value
*S. niger*	76	21	27.63	47	1.76	0.48	**<0.01**	76	23	30.2	30	1.30	0.39	**<0.01**
*S. esocinus*	77	14	18.18	28	2.0	0.36	**<0.01**	73	22	30.13	46	2.09	0.63	**<0.01**
*S. curvifrons*	71	12	16.9	21	1.75	0.29	**<0.01**	71	30	42.25	49	1.63	0.69	**>0.05**

*Total*	*224*	*47*	*20.98*	*96*	*2.04*	*0.42*	*<0.01*	*220*	*75*	*34.09*	*125*	*1.66*	*0.56*	*<0.01*

**Table 6:** Infection dynamics of *Pomphorhynchus kashmirensis* recorded of *Schizothorax* spp. from Dal Lake and River Jhelum.

Season	Host	Dal Lake							River Jhelum						
		Number examined	Number infected	Prevalence (%)	Number of parasites	Mean intensity	Abundance	*p* value	Number examined	Number infected	Prevalence (%)	Number of parasites	Mean intensity	Abundance	*p* value
Spring	*S. niger*	14	6	42.85	14	2.33	1	>0.05	15	4	26.6	12	3	0.8	>0.05
	*S. esocinus*	19	3	15.78	3	1	0.05	<0.01	20	5	25	8	1.6	0.4	<0.05
	*S. curvifrons*	16	2	12.5	6	3	0.18	<0.01	19	9	47.3	6	0.6	0.31	>0.05

Summer	*S. niger*	18	7	38.8	9	1.28	0.5	>0.05	16	8	50	7	0.87	0.43	>0.05
	*S. esocinus*	17	5	29.4	13	2.6	0.76	>0.05	18	8	44.4	15	1.87	0.83	>0.05
	*S. curvifrons*	19	4	21.05	6	1.5	0.31	<0.05	15	9	60	18	2	1.2	>0.05

Autumn	*S. niger*	25	6	24	13	2.16	0.52	<0.05	20	8	40	7	0.87	0.35	>0.05
	*S. esocinus*	19	4	21.05	9	2.25	0.47	<0.05	19	7	36.84	12	1.71	0.63	>0.05
	*S. curvifrons*	18	5	27.7	7	1.4	0.38	>0.05	22	9	40.9	14	1.55	0.63	>0.05

Winter	*S. niger*	19	2	10.5	11	5.5	0.57	<0.01	25	3	12	4	1.33	0.16	<0.01
	*S. esocinus*	22	2	9.09	3	1.5	0.13	<0.01	16	2	12.5	11	5.5	0.68	<0.01
	*S. curvifrons*	18	1	5.55	2	2	0.11	<0.01	15	3	20	11	3.6	0.73	<0.05

	*Total*	*224*	*47*	*20.98*	*96*	*2.04*	*0.42*	*<0.01*	*220*	*75*	*34.09*	*125*	*1.66*	*0.56*	*<0.01*

**Table 7:** Gender-wise infection dynamics of *Pomphorhynchus kashmirensis* recorded of* Schizothorax* spp. from Dal Lake and River Jhelum.

Season	Host	Dal Lake							River Jhelum						
		Number examined	Number infected	Prevalence (%)	Number of parasites	Mean intensity	Abundance	*p* value	Number examined	Number infected	Prevalence (%)	Number of parasites	Mean intensity	Abundance	*p* value
*S. niger*	Male	43	16	37.2	28	1.75	0.65	<0.05	33	12	36.36	22	1.83	0.66	<0.05
	Female	33	5	15.5	19	3.8	0.57		43	11	25.58	8	0.72	0.18	

*S. esocinus*	Male	39	8	20.5	15	1.8	0.38	>0.05	47	14	29.78	26	1.8	0.55	>0.05
	Female	38	6	15.7	13	2.16	0.34		26	8	30.76	20	2.5	0.76	

*S. curvifrons*	Male	52	7	13.46	15	2.14	0.28	>0.05	37	16	43.24	32	2	0.86	>0.05
	Female	19	5	26.3	6	1.2	0.31		34	14	41.17	17	1.2	0.5	

**Table 8:** Overall prevalence of *Bothriocephalus acheilognathi *in various host species.

Host	Dal Lake							River Jhelum						
	Number examined	Number infected	Prevalence (%)	Number of parasites	Mean intensity	Abundance	*p* value	Number examined	Number infected	Prevalence (%)	Number of parasites	Mean intensity	Abundance	*p* value
*S. niger*	76	22	28.94	153	6.9	2.01	<0.01	76	26	34.21	202	7.7	2.65	<0.01
*S. esocinus*	77	24	31.16	139	5.7	1.80	<0.05	73	25	34.24	144	5.7	1.97	<0.05
*S. curvifrons*	71	17	23.94	80	4.7	1.12	<0.01	71	22	30.98	155	7	2.18	<0.01

*Total*	*224*	*63*	*28.12*	*372*	*5.9*	*1.66*	*<0.01*	*220*	*73*	*33.18*	*501*	*6.8*	*2.27*	*<0.01*

**Table 9:** Infection dynamics of *Bothriocephalus acheilognathi *recorded of *Schizothorax *spp. from Dal Lake and River Jhelum.

Season	Host	Dal Lake							River Jhelum						
		Number examined	Number infected	Prevalence (%)	Number of parasites	Mean intensity	Abundance	*p* value	Number examined	Number infected	Prevalence (%)	Number of parasites	Mean intensity	Abundance	*p* value
Spring	*S. niger*	14	9	64.28	80	8.8	5.71	>0.05	15	8	53.33	56	7	3.73	>0.05
	*S. esocinus*	19	7	36.84	56	8	2.94	>0.05	20	7	35	48	6.8	2.4	>0.05
	*S. curvifrons*	16	7	43.75	42	6	2.62	>0.05	19	5	26.31	35	7	1.84	>0.05

Summer	*S. niger*	18	6	33.33	38	6.3	2.11	>0.05	16	9	56.25	82	9.1	5.12	>0.05
	*S. esocinus*	17	7	41.17	9	1.2	0.52	>0.05	18	8	44.44	36	4.5	2	>0.05
	*S. curvifrons*	19	4	21.05	12	3	0.63	<0.05	15	6	40	28	4.6	1.86	>0.05

Autumn	*S. niger*	25	3	12	6	2	0.24	<0.01	20	5	25	55	11	2.75	<0.05
	*S. esocinus*	19	5	26.31	35	7	1.84	>0.05	19	7	36.84	48	6.8	2.52	>0.05
	*S. curvifrons*	18	4	22.22	17	4.2	0.94	<0.05	22	8	36.36	74	9.2	3.36	>0.05

Winter	*S. niger*	19	4	21.05	29	7.2	1.52	<0.05	25	4	16	9	2.2	0.36	<0.01
	*S. esocinus*	22	5	22.72	39	7.8	1.77	<0.05	16	3	18.75	12	4	0.75	<0.05
	*S. curvifrons*	18	2	11.11	9	4.5	0.5	<0.05	15	3	20	18	6	1.2	<0.05

	*Total*	*224*	*63*	*28.12*	*372*	*5.9*	*1.66*	*<0.01*	*220*	*73*	*33.18*	*501*	*6.8*	*2.27*	*<0.01*

**Table 10:** Gender-wise infection dynamics of *Bothriocephalus acheilognathi* recorded of *Schizothorax* spp. from Dal Lake and River Jhelum.

Season	Host	Dal Lake							River Jhelum						
		Number examined	Number infected	Prevalence (%)	Number of parasites	Mean intensity	Abundance	*p* value	Number examined	Number infected	Prevalence (%)	Number of parasites	Mean intensity	Abundance	*p* value
*S. niger*	Male	43	13	30.23	87	6.6	2.3	>0.05	33	17	51.51	123	7.2	3.72	<0.01
	Female	33	9	27.27	66	7.3	1.9		43	9	20.93	79	8.7	1.83	

*S. esocinus*	Male	39	16	41.02	97	6	2.1	>0.05	47	19	40.42	92	4.8	1.95	>0.05
	Female	38	8	21.05	42	5.2	1.9		26	6	23.07	52	8.6	2	

*S. curvifrons*	Male	52	13	25	67	5.1	2	>0.05	37	12	32.43	108	9	2.91	>0.05
	Female	19	4	21.05	13	3.2	0.4		34	10	29.41	47	4.7	1.38	

**Table 11:** Overall prevalence of *Adenoscolex oreini *in various host species.

Host	Dal Lake							River Jhelum						
	Number examined	Number infected	Prevalence (%)	Number of parasites	Mean intensity	Abundance	*p* value	Number examined	Number infected	Prevalence (%)	Number of parasites	Mean intensity	Abundance	*p* value
*S. niger*	76	24	31.57	95	3.95	1.25	<0.01	76	22	28.94	180	8.18	2.36	<0.01
*S. esocinus*	77	22	28.57	145	6.59	1.88	<0.01	73	24	32.87	182	7.58	2.49	<0.01
*S. curvifrons*	71	25	35.21	131	5.24	1.84	<0.05	71	27	38.02	183	6.77	2.57	>0.05

*Total*	*224*	*71*	*31.69*	*371*	*5.22*	*1.65*	*<0.01*	*220*	*73*	*33.18*	*545*	*7.46*	*2.47*	*<0.01*

**Table 12:** Infection dynamics of *Adenoscolex oreini* recorded of *Schizothorax* spp. from Dal Lake and River Jhelum.

Season	Host	Dal Lake							River Jhelum						
		Number examined	Number infected	Prevalence (%)	Number of parasites	Mean intensity	Abundance	*p* value	Number examined	Number infected	Prevalence (%)	Number of parasites	Mean intensity	Abundance	*p* value
Spring	*S. niger*	14	6	42.85	29	4.8	2.07	>0.05	15	5	33.33	36	7.2	2.4	>0.05
	*S. esocinus*	19	5	26.31	14	2.8	0.73	>0.05	20	8	40	54	6.7	2.7	>0.05
	*S. curvifrons*	16	7	43.75	38	5.4	2.37	>0.05	19	7	36.84	35	5	1.84	>0.05

Summer	*S. niger*	18	9	50	42	4.6	2.33	>0.05	16	6	37.5	80	13.3	5	>0.05
	*S. esocinus*	17	8	47.05	92	11.5	5.41	>0.05	18	7	38.88	42	6	2.33	>0.05
	*S. curvifrons*	19	9	47.36	56	6.2	2.94	>0.05	15	9	60	39	4.3	2.6	>0.05

Autumn	*S. niger*	25	6	24	18	3	0.72	<0.05	20	7	35	52	7.4	2.6	>0.05
	*S. esocinus*	19	5	26.31	27	5.4	1.42	>0.05	19	6	31.57	65	10.8	3.42	>0.05
	*S. curvifrons*	18	7	38.88	29	4.1	1.61	>0.05	22	7	31.81	80	11.4	3.63	>0.05

Winter	*S. niger*	19	3	15.78	6	2	0.31	<0.01	25	4	16	12	3	0.48	<0.01
	*S. esocinus*	22	4	18.18	12	3	0.54	<0.01	16	3	18.75	21	7	1.31	<0.05
	*S. curvifrons*	18	2	11.11	8	4	0.44	<0.01	15	4	26.66	29	7.2	1.93	>0.05

	*Total*	*224*	*71*	*31.69*	*371*	*5.2*	*1.65*	*<0.01*	*220*	*73*	*33.18*	*545*	*7.4*	*2.47*	*<0.01*

**Table 13:** Gender-wise infection dynamics of *Adenoscolex oreini *recorded of *Schizothorax* spp. from Dal Lake and River Jhelum.

Season	Host	Dal Lake							River Jhelum						
		Number examined	Number infected	Prevalence (%)	Number of parasites	Mean intensity	Abundance	*p* value	Number examined	Number infected	Prevalence (%)	Number of parasites	Mean intensity	Abundance	*p* value
*S. niger*	Male	43	16	37.20	63	3.9	1.46	>0.05	33	14	42.42	101	7.2	3.06	<0.05
	Female	33	8	24.24	32	4	0.96		43	8	18.60	79	9.8	1.83	

*S. esocinus*	Male	39	13	33.33	97	7.4	2.48	>0.05	47	17	36.17	120	7	2.55	>0.05
	Female	38	9	23.68	48	5.3	1.26		26	7	26.92	62	8.8	2.38	

*S. curvifrons*	Male	52	15	28.84	94	6.2	1.80	>0.05	37	12	32.43	125	10.4	3.37	>0.05
	Female	19	10	52.63	37	3.7	1.94		34	9	26.47	58	6.4	1.70	

**Table 14:** Correlation between environmental variables and prevalence of *Pomphorhynchus Kashmirensis*, *Adenoscolex oreini*, and *Bothriocephalus acheilognathi*.

Environmental variables	Prevalence of *Pomphorhynchus kashmirensis*				Prevalence of *Adenoscolex oreini*				Prevalence of *Bothriocephalus acheilognathi*			
	Dal Lake		River Jhelum		Dal Lake		River Jhelum		Dal Lake		River Jhelum	
	Pearson's coef.	Spearman's rho	Pearson's coef.	Spearman's rho	Pearson's coef.	Spearman's rho	Pearson's coef.	Spearman's rho	Pearson's coef.	Spearman's rho	Pearson's coef.	Spearman's rho
Temperature	.882^*∗∗*^	.853^*∗∗*^	.907^*∗∗*^	.907^*∗∗*^	.802^*∗∗*^	.699^*∗*^	.810^*∗∗*^	.592^*∗*^	.722^*∗∗*^	.664^*∗*^	.922^*∗∗*^	.729^*∗∗*^
Oxygen	−.842^*∗∗*^	−.825^*∗∗*^	−.451	−.350	−.755^*∗∗*^	−.643^*∗*^	−.105	−.084	−.297	−.280	.017	.056
pH	.420	.587^*∗*^	−.227	−.266	.487	.601^*∗*^	−.111	−.182	.231	.231	.182	.364
Carbon dioxide	.682	.021	.628^*∗*^	.522	−.007	−.035	.529	.322	.598^*∗*^	.629^*∗*^	.579^*∗*^	.581^*∗*^

^*∗*^Correlation is significant at the 0.05 level (2-tailed).

^*∗∗*^Correlation is significant at the 0.01 level (2-tailed).

## Abbreviations

*P. kashmirensis*: * Pomphorhynchus kashmirensis*
*B. acheilognathi*: * Bothriocephalus acheilognathi*
* A. oreini*: * Adenoscolex oreini*
* S. niger*: * Schizothorax niger*
* S. esocinus*: * Schizothorax esocinus*
* S. curvifrons*: * Schizothorax curvifrons*.
